# Risk factors affecting seroconversion after influenza A/H1N1 vaccination in hemodialysis patients

**DOI:** 10.1186/1471-2369-13-165

**Published:** 2012-12-03

**Authors:** Sung Jin Moon, Sang Hun Lee, Young-Ho Byun, Gi Young Yun, Seung Kyu Kim, Baik-Lin Seong, Ah Reum Kim, Eun sun Park, Hyung-Jong Kim, Jung Eun Lee, Sung Kyu Ha, Jae Myun Lee, Hyeong-Cheon Park

**Affiliations:** 1 Myongji Hospital, College of Medicine, Kwandong University, Goyang, Korea; 2 Department of Internal Medicine, Gangnam Severance Hospital, Institute of Vascular and Metabolism Research, College of Medicine, Yonsei University, 146-92 Dogok-Dong, Gangnam-Gu, Seoul 135-720, Korea; 3 Department of Biotechnology, College of Life Science & Biotechnology, Yonsei University, Seoul, Korea; 4 Department of Microbiology and Brain Korea 21 Project for Medical Sciences, Institute for Immunology and Immunological Diseases, Yonsei University College of Medicine, 134, Shinchon-dong, Seodaemoon-gu 120-752 Seoul, Korea; 5 BundangCHA Medical Center, CHA University, Sungnam, Korea; 6 Yongin Severance Hospital, College of Medicine, Yonsei University, Yongin, Korea

**Keywords:** Hemodialysis, Pandemic H1N1/2009 influenza, Vaccine, Seroconversion

## Abstract

**Abstracts:**

**Background**

Hemodialysis (HD) patients have multiple causes of immune dysfunction and poor immune response to influenza vaccination. We investigated the antibody response rate to a pandemic H1N1/2009 influenza vaccination and clinical parameters influencing the induction of antibody responses in HD patients.

**Methods**

A total of 114 HD patients were vaccinated with a monovalent adjuvanted H1N1 inactivated influenza vaccine. Titers of neutralizing antibodies were evaluated by hemagglutination inhibition (HI) assay at pre- and 4 weeks after vaccination. Seroconversion was defined as either a pre-vaccination HI titer < 1:10 and a post vaccination HI titer > 1:40 or a pre-vaccination HI titer ≥ 1:10 and a minimum four-fold rise in post-vaccination HI antibody titer. Seventeen out of 114 HD patients (14.9%) tested positive for antibodies against influenza A/H1N1/2009 before vaccination. The remaining 97 baseline sero-negative patients were included in the analysis.

**Results**

Only 30 (30.9%) HD patients had seroconversion 4 weeks after vaccination. The elderly patients, those over 65 years of age, showed significantly lower seroconversion rate compared to younger HD patients (20.5% vs. 39.6%, p = 0.042). Furthermore, patients with hemoglobin values less than 10 g/dL had a significantly lower seroconversion rate compared to those with higher hemoglobin values (20.0 vs. 38.6%, p = 0.049). By multivariate logistic regression analysis, only age ≥65 years (OR = 0.336, 95% confidence interval (CI) 0.116-0.971, p = 0.044) and hemoglobin levels <10 g/dL (OR = 0.315, 95% CI 0.106-0.932, p = 0.037) were independently associated with seroconversion after vaccination.

**Conclusions**

Our data show that HD patients, especially who are elderly with low hemoglobin levels, are at increased risk for lower seroconversion rate after influenza A/H1N1 vaccination. Further studies are needed to improve the efficacy of vaccination in these high risk patients.

## Background

End stage renal disease (ESRD) patients have complex multifactorial causes of immune dysfunction and are at high risk for influenza infection and its complications. Following the outbreak of pandemic influenza A/H1N1 in 2009, the World Health Organization (WHO) and the Korean Food and Drug Administration recommended vaccination of pandemic influenza A/H1N1 vaccine for all high risk individuals, including ESRD patients on hemodialysis (HD) [1,2]. However, the current recommendations for these high risk patients are based on data from clinical trials performed on healthy subjects, in which a single-dose vaccine provided a sufficient antibody seroconversion rate of 80 to 96% [2]. Compared with the general population, HD patients have poor immune responses to seasonal influenza vaccination [3]. Although mass vaccination for the pandemic influenza A/H1N1 has been implemented in high risk groups, specific antibody responses after vaccination are lacking in HD patients. Therefore, the purpose of the present study is to investigate the antibody response rate to a single-dose inactivated, pandemic H1N1 influenza vaccination and establish possible clinical and biochemical parameters that may influence the induction of antibody responses in HD patients.

## Patients and methods

### Study design

This multicenter, prospective observational cohort study was conducted from December 2009 to March 2010. Participating clinical sites included hemodialysis unit from Gangnam Severance hospital, Bundang CHA Medical Center, Yongin Severance Hospital, and three private dialysis clinics. Clinically stable HD patients were invited to participate in the study during their routine hemodialysis treatments. Patients with fever (temperature >38°C) or flu-like symptoms, age less than 18 years old, known allergy to egg proteins, any hospitalization within three months, liver diseases, malignancy, or treatment with immunosuppressive drugs were excluded. A total of 114 HD patients on hemodialysis thrice a week with synthetic membranes for more than 3 months were enrolled. The causes of ESRD were diabetic nephropathy (n = 62, 63.9%), hypertension (n = 18, 18.9%), chronic glomerulonephritis (n = 6, 6.2%), polycystic kidney disease (n = 5, 5.2%), and unknown origin (n = 6, 6.2%). The patients with unknown origin had no clinical characteristics or lab findings (ANA, complements, ANCA, anti-GBM antibody, etc.) of autoimmune diseases or immune-complex glomerulonephritis. This study was approved by the Institutional Review Board Committee of Gangnam Severance Hospital (3-2009-0170). After informed consent was obtained from the patients, baseline blood samples were taken before the midweek dialysis for the determination of baseline hemagglutinin (HA) antibody titers, and the monovalent adjuvanted (MF59C1) H1N1 inactivated influenza vaccine (Green-Flu-S plus®3.75ug/0.25 ml, Green Cross Co. Ltd., Yongin, Korea) was injected intramuscularly. Four weeks after vaccination, blood samples were collected again for the assessment of post-vaccination HA antibody titers.

### Hemagglutination inhibition (HI) assay

The titers of neutralizing antibodies to pandemic influenza virus were evaluated by HI assay according to the standard WHO procedure with influenza A/Seoul/Y-01/2009 virus [4].The sera were treated with receptor destroying enzyme (RDE, Denka Seiken Co. Ltd., Tokyo, Japan) by diluting 7 ul of serum with 21 ul RDE and were incubated overnight at 37°C. The enzyme was inactivated by 1 hr incubation at 56°C followed by addition of 42 ul of PBS for a final dilution of 1:10. Virus suspensions containing 4 HA units of virus were incubated for 1 h with serial 2-fold dilutions of antiserum. HI assays were performed in V-bottom 96-well microtiter plates with 1.0% chicken erythrocytes. HI titer was determined as the reciprocal of the highest dilution that showed complete inhibition of hemagglutination. Seroconversion was defined as either a pre-vaccination HI titer < 1:10 and a post vaccination HI titer > 1:40 or a pre-vaccination HI titer ≥ 1:10 and a minimum four-fold rise in post-vaccination HI antibody titer.

### Statistics

All statistical analyses were performed using SPSS for Windows version 17.0 software (SPSS, Inc, an IBM Company, Chicago, Illinois, USA). Values were expressed mean ± standard deviation (SD) or percentages. Differences between two groups according to the immune response after vaccination were analyzed by t-test in continuous variables or Chi-square test in dichotomous variables. Non-parametric variables were log-transformed before multivariate analysis. Binary logistic analysis was used for identifying independent parameters associated with seroconversion after the influenza A/H1N1vaccination. It was adjusted by significant variables in the univariate analysis (p <0.15) and known associated factors for HD patients [diabetes mellitus (DM), albumin, ferritin levels and urea reduction rate]. Null hypotheses of no difference were rejected if p-values were less than .05.

## Results

There were 200 eligible HD patients, and 86 patients declined to participate. The remaining 114 patients were enrolled in the study and 17 out of 114HD patients (14.9%) tested positive for influenza A/H1N1/2009 HA specific antibodies at baseline before vaccination. There were no significant differences in demographic or biochemical parameters between the patients who accepted and refused to participate, and between pre-vaccination HA antibody positive and negative patients (data not shown). The remaining 97 baseline sero-negative patients were thus included in the final analysis (male 54, mean age was 60.9 ± 12.6 years) for the study. Among the 97 pre-vaccination antibody-negative patients, only 30 (30.9%) had seroconversion (responders: ≥4-fold increase in titer) 4 weeks after influenza A/H1N1 vaccination. The patients’ demographic, biochemical parameters, dialysis adequacy and medications are shown in Table 1. Demographic features such as gender, body weight, presence of DM, iron status reflected by serum ferritin, and parathyroid hormone were not associated with seroconversion rate.

**Table 1:** Baseline characteristics of the patients according to the seroconversion or not

**Variables**	**All patients (n = 97)**	**Nonresponders (n = 67)**	**Responders (n = 30)**	**P-value**
Age (years)	60.9 ± 12.6	63.1 ± 12.0	56.3 ± 12.6	0.012
Sex (Male,%)	54 (56.7)	35 (53.0)	19 (61.3)	0.445
Diabetes Mellitus (%)	62 (63.9)	44 (65.7)	18 (60.0)	0.591
Body mass index (Kg/m^2^)	22.7 ± 3.5	22.7 ± 3.6	22.8 ± 3.4	0.898
Duration of dialysis (mo)*	41.0 (25–41)	37.5 (22.8-66.3)	53.0 (31.0-82.0)	0.082
KT/V	1.44 ± 0.32	1.44 ± 0.33	1.41 ± 0.28	0.705
Urea reduction rate (%)	69.3 ± 7.9	70.0 ± 7.8	67.8 ± 8.2	0.193
Hepatitis B or C (%)	8 (8.2)	5 (7.5)	3 (10.0)	0.708
Medications (%)				
Angiotensin converting enzyme inhibitor	18 (18.6)	11 (16.4)	7 (23.3%)	0.418
Angiotensin II receptor blocker	60 (61.9)	37 (55.2)	23 (76.7)	0.044
Calcium channel blocker	60 (61.9)	41 (61.2)	19 (63.3)	0.841
Beta blocker	49 (50.5)	30 (44.8)	19 (63.3)	0.194
Diuretics	53 (54.6)	38 (56.7)	15 (50.0)	0.539
Statins	40 (42.3)	28 (41.8)	12 (40.0)	0.868
Vitamin D analogue	5 (5.2)	4 (6.0)	1 (3.3)	0.587
White blood cell (/mm^3^)	6,450 ± 1,846	6,490 ± 1,977	6,368 ± 1,561	0.765
Lymphocyte count (/mm^3^)	1,376 ± 547	1,432 ± 568	1,225 ± 479	0.313
Hemoglobin (g/dL)	10.2 ± 1.1	10.0 ± 1.14	10.5 ± 0.85	0.032
MCV (fL)	93.7 ± 5.1	94.4 ± 5.3	91.4 ± 3.6	0.075
MCH (pg)	30.9 ± 1.7	31.1 ± 1.7	30.4 ± 1.3	0.153
MCHC (g/dL)	33.1 ± 1.1	33.0 ± 1.1	33.2 ± 1.1	0.546
Ferritin (ng/mL)*	159 (107–255)	159 (98–255)	155 (108–259)	0.367
C-reactive protein (mg/L)*	1.1 (0.5-4.7)	1.1 (0.5-4.3)	2.5 (0.45-6.1)	0.446
Glucose (mg/dL)	133 ± 48	134 ± 48	130 ± 48	0.74
Uric acid (mg/dL)	7.3 ± 1.3	7.2 ± 1.3	7.4 ± 1.4	0.241
Total Cholesterol (mg/dL)	149 ± 35	153 ± 40	149 ± 27	0.477
Albumin (g/dL)	4.0 ± 0.4	4.0 ± 0.4	4.0 ± 0.3	0.874
Alkaline phosphatase (IU/L)	91 ± 55	84 ± 50	101 ± 62	0.170
Total bilirubin (mg/dL)	0.31 ± 0.14	0.32 ± 0.15	0.29 ± 0.12	0.422
Triglyceride (mg/dL)	129 ± 94	133 ± 106	117 ± 60	0.351
HDL-cholesterol (mg/dL)	38 ± 13	37 ± 13	41 ± 14	0.332
Intact-parathyroid hormone (pg/mL)	160 ± 97	148 ± 96	188 ± 97	0.069

* was expressed by median (interquartile range).

**Figure 1:**
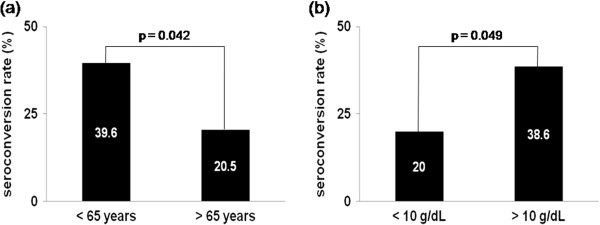
The differences of seroconversion rate according to the age 65 years (a) and hemoglobin 10 g/dL (b).

HD patients who had seroconversion 4 weeks post-vaccination were significantly younger (56.3 ± 12.6 vs. 63.1 ± 12.0 years, p = 0.012) and had higher hemoglobin values (10.5 ± 0.85 vs. 10.0 ± 1.14 g/dL, p = 0.032) than those who failed to show seroconversion (nonresponders). In particular, the elderly patients, those over 65 years of age, had significantly lower seroconversion rates compared to younger HD patients (20.5 vs. 39.6%, p = 0.042, Figure 1). In addition, patients with hemoglobin values greater than 10 g/dL had significantly higher seroconversion rates compared to those with less than hemoglobin values (38.6 vs. 20.0%, p-value = 0.049). Patients were further divided into 4 groups by hemoglobin level and age. (Group 1: Hb ≥ 10 g/dL and Age < 65 years, Group 2: Hb ≥ 10 g/dL and Age ≥ 65 years, Group 3: Hb < 10 g/dL and Age < 65 years, Group 4: Hb < 10 g/dL and Age ≥ 65 years). Group 4 showed a significantly lower seroconversion rate compared with Group 1 (10.0% vs 45.5%, p = 0.014). Logistic regression analysis for seroconversion showed significantly lower odds ratio for Group 4 (OR 0.133 (0.027-0.669), p = 0.014) (Table 2).

**Table 2:** Odds ratio for seroconversion by hemoglobin level 10 g/dL and age 65 years by logistic regression analysis

**Patients group**	**Odds ratio**	**95% CI**	**p-value**
Group 1 (Hb ≥ 10 g/dL and Age < 65 years)	1.0 (reference)		
Group 2 (Hb ≥ 10 g/dL and Age ≥ 65 years)	0.494	0.162-1.508	0.215
Group 3 (Hb < 10 g/dL and Age < 65 years)	0.514	0.159-1.668	0.268
Group 4 (Hb < 10 g/dL and Age ≥ 65 years)	0.133	0.027-0.669	0.014

Other variables specific for hemodialysis such as dialysis dosage measured by Kt/V or urea reduction rate and dialysis vintage were not associated with seroconversion rate.

By logistic regression analysis adjusted by age, hemoglobin, DM, angiotensin II receptor blocker usage, serum albumin, ferritin, intact parathyroid hormone, urea reduction rate, and log dialysis vintage, only age older than 65 years (OR = 0.336, 95% confidence interval (CI) 0.116-0.971, p = 0.044) and hemoglobin levels less than 10 g/dL (OR = 0.315, 95% CI 0.106-0.932, p = 0.037) were independently associated with seroconversion after vaccination in HD patients (Table 3).

**Table 3:** Multiple logistic regression analysis of risk factors associated with seroconversion

	**Univariate**		**Multivariate**	
**Variables**	**Odds ratio**	**95% CI**	**Odds ratio**	**95% CI**
Age (vs. < 65 year)	0.392	0.157-0.980	0.336	0.116-0.971
Hemoglobin (vs. ≥10 g/dL)	0.370	0.145-0.944	0.315	0.106-0.932
Diabetes mellitus (vs. non-DM)	0.784	0.323-1.905	0.912	0.319-2.604
ARB use (vs. non-ARB use)	2.664	1.006-7.052	1.785	0.597-5.333
Albumin (g/dL)	0.881	0.286-2.712	0.294	0.069-1.255
Ferritin (ng/mL)	1.002	0.999-1.004	1.002	0.999-1.005
Intact parathyroid hormone (pg/mL)	1.004	1.000-1.009	1.005	1.000-1.010
Urea reduction rate (%)	0.969	0.915-1.026	0.954	0.892-1.020
log dialysis duration	3.093	0.885-10.808	3.160	0.805-12.410

ARB, angiotensin II receptor blocker.

adjusted by age, hemoglobin, diabetes mellitus, angiotensin II receptor blocker, serum albumin, ferritin, intact parathyroid hormone, urea reduction rate, and log dialysis duration.

Except for mild to moderate local pain at the injection site, no other serious side effects associated with the vaccination were observed in HD patients.

## Discussion and conclusions

Our prospective observational cohort study investigated the seroconversion rate to monovalent adjuvanted H1N1 inactivated influenza vaccine in clinically stable HD patients. The rate of seroconversion was 30.9% for all HD patients. It is even lower than the previously reported seroconversion rate for Korean diabetic patients by our group and much lower than the one reported for normal controls (68.1 ~ 84.4%) using the same vaccine [2,5]. The reported seroconversion rate after influenza A/H1N1 vaccination is quite variable, from 33.3% to 64.2%, with contrasting results [6,7]. Compared with the healthy controls, HD patients have a lower seroconversion rate and an inability to maintain adequate antibody titers over time [8-10]. The mechanism of uremia-associated hyporesponsiveness to vaccination is still incompletely understood.

Over the past decade, a large number of studies have shown that a variety of vaccines are less efficient in elderly persons [11]. A clear association between older age and poor immune response to HBV vaccine was shown in recent meta-analysis in ESRD patients [12]. Similarly, other studies showed that age significantly affected immune response to influenza A (H1N1) vaccine in general population [13,14]. Goodwin et al. reported that after adjusting for vaccine and host factors, vaccine response in the elderly was approximately 1/4 as rigorous for H1 and B antigens and about 1/2 as rigorous for H3 antigens, compared to the antibody response in younger adults [15]. The seroconversion rate of the elderly patients in the present study was no more than 20.5%. In the multivariate logistic regression analysis, age over 65 years was a significant risk factor for hyporesponsiveness to influenza vaccine. The mechanisms for age related change in the antibody response are not fully understood, however, memory CD4^+^ T cell impairment and imbalance in the production of Th1 and Th2 cytokines are considered to be related with the lower antibody response [16,17].

In contrast, there are some studies that did not observe “age factors” influencing antibody response to influenza vaccines [18,19]. The discrepancies in the different studies’ results may be explained by differences in types of vaccines used, use of adjuvants, host immune factors, and previous vaccinations [3].

A recent large cohort trial (n = 355) employing one dose schedule of pandemic H1N1 vaccine demonstrated encouraging immunogenic profile in both adults and elderly age-group [20]. While it is still controversial, two doses of a pandemic vaccine to elicit an improved protective immune response has been recommended in high risk immunocompromised patients [21-23]. Our vaccination protocol consisted of one dose of intramuscular injection of monovalent adjuvanted (MF59C1) H1N1 inactivated influenza vaccine. The vaccine used in our trial fulfilled all international licensing criteria in both the adult and elderly age-groups [24,25]. However, it elicited lower immune response in elderly age-group (over 65 years of age) compared to those of younger adults (37.4 ~ 53.5% vs. 68.1 ~ 84.4%, respectively). This immune response was significantly improved after a second dose of vaccine administration (76 ~ 91%) [2]. Therefore, it may be prudent to measure baseline and post-vaccination protective antibody titers against the pandemic H1N1strain in high risk patients. Our results provide alarming information on low immune response to pandemic influenza vaccination in elderly HD patients and further clinical trials administering second or booster doses to improve seroconversion rates in high risk patients may be warranted.

In contrast to other studies [7,10,26], hemoglobin level less than 10 g/dL was a significant risk factor for impaired seroconversion in the present study. The relationship between anemia and poor antibody responsiveness after vaccination is an open question. However, anemia is commonly associated with inflammation and nutritional status of HD patients [27]. There are several studies that reported inflammation and nutritional status as important risk factors of the hypo-responsiveness after vaccination. Fulop et al. observed that non-response group after influenza vaccination in the elderly patients had lower nutritional parameters such as hemoglobin, total protein, iron, vitamin E, and DHEA [28]. Recent research by Chang et al. also suggested that hemoglobin level is a significant predictor for seroresponse and seroconversion in HD patients [19]. Our study did not have enough power to assess the influence of inflammatory or nutritional parameters on seroconversion. However, considering these relationship, we suggest that anemia may be a risk factor for hypo-responsiveness after vaccination. Furthermore, when the patients were divided into 4 groups by hemoglobin level and age, Group 4 (Hb < 10 g/dL and Age ≥ 65 years) had a significant lower seroconversion rate and odds ratio for seroconversion compared with Group 1 (Hb ≥ 10 g/dL and Age < 65 years). Group 2 and 3 did not differ from the reference Group 1. These results show that combined age and anemia, defined as hemoglobin level less than 10 g/dL, are significant additional risk factors for seroconversion after influenza vaccination.

Other factors influencing seroconversion have been investigated in several trials but no definite common factor has been identified. Some investigators reported that very high ferritin levels may suppress antibody production following influenza vaccination in HD patients [29], but our results and other investigators have not found any definite association between iron status and seroconversion rate.

Recently, a weaker immune response to non-adjuvanted 2009 influenza A (H1N1) vaccine (Panenza®; Sanofi Pasteur, France) was reported in HD patients dialyzed twice a week whereas other HD patients undergoing dialysis 3 times a week showed comparable immune response to healthy controls [26].However, their results should be interpreted with caution as half of the study participants were underdialyzed and non-adjuvanted vaccine was used for vaccination. All of our patients underwent hemodialysis treatment 3 times a week and the achieved dialysis dose and time on dialysis were comparable between responders and non-responders. Moreover, most of other studies evaluating the efficacy of influenza A H1N1/2009 vaccine using adjuvanted vaccine have shown that dialysis dose or degree of uremia does not influence seroconversion [6,7].

Compared to non-diabetic patients, diabetic patients had an impaired immune response to influenza vaccination [30]. In our previous study on diabetic patients, only 48.6% of patients achieved seroconversion after a single-dose adjuvanted, inactivated, pandemic H1N1 influenza vaccination [5]. The hemodialysis patients in the present study, however, did not show significant effect of DM on the seroconversion rate. Furthermore, subgroup analysis according to age and gender showed no significant effect of DM on seroconversion (data not shown). This observed discrepancy may be explained by several causes. First, age of our seroresponders in diabetic patients was younger compared to that of nonresponders (57.5 ± 11.1 vs. 65.1 ± 11.5 years, p = 0.021). Advanced age negatively affects the seroconvesion rate regardless of the patient’s status [12,31]. Second, uremic milieu per se is commonly believed to be the cause of poor seroconversion in ESRD patients. Our results are in accord with recent study from Taiwan, which reported no significant differences between DM and non-DM HD patients [19]. Another possible explanation might be the seroconversion rate of our patients was very low (30.8%) and could have undermined the effect of DM. Non-diabetic HD patients had a similarly poor seroconversion rate as their diabetic counterparts, suggesting that state of uremia per se is associated with substantial immunodeficiency.

There are several limitations in this study. First, healthy controls were not included in the study. Therefore, we could not compare the seroconversion rate between HD patients and healthy controls. However, the seroconversion rate for healthy Korean individuals using the same adjuvanted vaccine is 68.1%, clearly greater than our HD patients. Second, the number of patients is small and previous vaccination history was not surveyed. Nevertheless, the efficacy of influenza vaccination in the present study is relatively consistent with other studies [6]. Third, long-term immunogenicity of influenza vaccination was not investigated in our study. Short term immune responses to influenza vaccination have been well studied but long-term immune response in HD patients are lacking. Long-term immunogenicity results would be more helpful in directing immunization schedule for these high risk patients.

In conclusion, we found that administration of a single dose adjuvanted monovalent H1N1 inactivated influenza vaccine induced relatively a poor immune response in HD patients. In particular the elderly HD patients with lower hemoglobin levels are at much increased risk. Further trials with larger number of patients and different vaccination schedules employing different vaccine doses or boosters are warranted.

## Competing interests

None of the authors has any competing interest.

## Authors’ contributions

SJM analyzed data and wrote the manuscript. SHL, YHB, and BLS collected and interpreted data. ARK and ESP performed the hemagglutinaion assay. HJK, JEL, and SKH managed the study and collected data. JML and HCP designed the study, analyzed data, and wrote the manuscript.GYY and SKK analyzed nonparticipants data and revised the manuscript. All authors read and approved the final manuscript.

## Pre-publication history

The pre-publication history for this paper can be accessed here:

http://www.biomedcentral.com/1471-2369/13/165/prepub
